# Cervical Cancer Neoantigen Landscape and Immune Activity is Associated with Human Papillomavirus Master Regulators

**DOI:** 10.3389/fimmu.2017.00689

**Published:** 2017-06-16

**Authors:** Yong Qin, Suhendan Ekmekcioglu, Marie-Andrée Forget, Lorant Szekvolgyi, Patrick Hwu, Elizabeth A. Grimm, Amir A. Jazaeri, Jason Roszik

**Affiliations:** ^1^Department of Melanoma Medical Oncology, The University of Texas MD Anderson Cancer Center, Houston, TX, United States; ^2^MTA-DE Momentum, Genome Architecture and Recombination Research Group, Department of Biochemistry and Molecular Biology, University of Debrecen, Debrecen, Hungary; ^3^Department of Gynecologic Oncology and Reproductive Medicine, The University of Texas MD Anderson Cancer Center, Houston, TX, United States; ^4^Department of Genomic Medicine, The University of Texas MD Anderson Cancer Center, Houston, TX, United States

**Keywords:** cervical cancer, neoantigens, human papillomavirus, master regulators, immunotherapy

## Abstract

Human papillomaviruses (HPVs) play a major role in development of cervical cancer, and HPV oncoproteins are being targeted by immunotherapies. Although these treatments show promising results in the clinic, many patients do not benefit or the durability is limited. In addition to HPV antigens, neoantigens derived from somatic mutations may also generate an effective immune response and represent an additional and distinct immunotherapy strategy against this and other HPV-associated cancers. To explore the landscape of neoantigens in cervix cancer, we predicted all possible mutated neopeptides in two large sequencing data sets and analyzed whether mutation and neoantigen load correlate with antigen presentation, infiltrating immune cell types, and a HPV-induced master regulator gene expression signature. We found that targetable neoantigens are detected in most tumors, and there are recurrent mutated peptides from known oncogenic driver genes (KRAS, MAPK1, PIK3CA, ERBB2, and ERBB3) that are predicted to be potentially immunogenic. Our studies show that HPV-induced master regulators are not only associated with HPV load but may also play crucial roles in relation to mutation and neoantigen load, and also the immune microenvironment of the tumor. A subset of these HPV-induced master regulators positively correlated with expression of immune-suppressor molecules such as PD-L1, TGFB1, and IL-10 suggesting that they may be involved in abrogating antitumor response induced by the presence of mutations and neoantigens. Based on these results, we predict that HPV master regulators identified in our study might be potentially effective targets in cervical cancer.

## Introduction

It is anticipated that most cervical cancer cases will be prevented in the future, but this disease is currently incurable and it causes around 4,000 deaths per year in the US (1). Low vaccination rates (2) forecast that cervical cancer prevalence and mortality will not decrease rapidly and novel treatments are also needed in addition to increasing vaccination uptake. Clinical trials show that immunotherapy of this malignancy is possible, and multiple new agents are currently being tested, including anti-CTLA-4, anti-PD-1, and anti-PD-L1 antibodies, and therapeutic vaccines as well (3). It is well known that human papillomaviruses (HPVs) cause the overwhelming majority of cervical tumors (4), and HPV proteins are attractive targets (5). Tumor-infiltrating lymphocytes specific to HPV E6 and E7 oncoproteins were successfully expanded and capable of tumor reactivity in an adoptive cell transfer trial in an autologous setting, however, only third of the patients responded in that study (6). *Listeria monocytogenes*-based immunotherapy is another promising approach showing specific activity against high-risk HPV strains (7). The importance of targeting non-viral antigens in HPV-driven cancers has also recently been demonstrated (8).

To increase efficacy of immunotherapies for the treatment of cervical cancer, we need a better understanding of the immune microenvironment of this malignancy. Employing an RNA sequencing-based metric of immune effector function, it was recently shown that immune cytolytic activity is high in cervical cancer compared to other tumors, and it is also higher in cervix tumor samples with a high mutation load (9). These observations suggest the presence of neoantigens derived from somatic mutations that are presented by human leukocyte antigen (HLA) class I molecules and attract cytotoxic T cells. However, as the immune system is unable to eliminate the cancer cells, suppressive mechanisms [such as regulatory T cells (Tregs), myeloid-derived suppressor cells (MDSCs), tumor-associated macrophages, or inhibitory cytokines (for example, IL-10, TGF-beta)] are probably also present in these high mutation burden tumors. To identify the key determinants of immune response or suppression, in the current study, we asked whether increased mutation burden [the number of non-silent exonic mutations, similarly how it was defined in earlier publications (10)] and neoantigen load (the number of mutated peptides derived from genes having non-zero expression that are predicted to bind the patient’s HLA) are associated with known antigen presentation and immune cell markers and subtypes.

Although it is known that HPV E6 and E7 oncoproteins play a major role in development of cervix cancer, other “driver pathways” that may also contribute to tumor progression also need to be identified and targeted to be able to successfully fight this disease (11). HPV16 E6 and E7 oncoprotein-related “master regulators” [EGR3, FOSB, NR4A2, PRDM1, SOX9, OVOL1, MNT, PA2G4, Enolase 1 (ENO1), TEAD4, FOXO4, and ZNF365], which have been shown to regulate multiple downstream effects of HPV16 and possibly other HPV types (12), are attractive candidate genes that could potentially regulate the immune microenvironment as well. Therefore, our goal was to also determine the association between expression of these regulators, antigen presentation, antitumor effector function, and immune suppression.

To achieve these goals, we used two large, publicly available cervical cancer data sets, namely the Cancer Genome Atlas (TCGA) cervical cancer data (13) and the exome and RNA sequencing data available in the publication of Ojesina et al. (14) to analyze the neoantigen landscape and the associated immune activity in cervical cancer. Our studies show that mutation load and neoantigen availability is associated with expression of HPV oncoprotein-associated “master regulators,” and also with specific genes involved in antigen presentation, immune cytotoxic T-cell function, and immunosuppressive mechanisms. The relationships we identified help to refine our knowledge on immune activity in cervical cancer and are intended to provide attractive targets to increase effectiveness of immunotherapies.

## Results

### Cervical Cancer Neoantigens Are Detectable and Potentially Immunogenic

**Figure 1:**
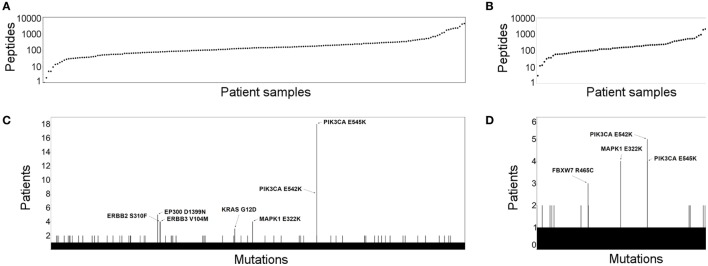
The neoantigen landscape and recurrent targets in cervical cancer. The number of predicted cervical cancer neoantigens is depicted for the Cancer Genome Atlas (TCGA) **(A)** and Ojesina et al. **(B)** data sets. Patient samples are shown in columns, where each dot represents the number of neopeptides predicted to bind human leukocyte antigen class I. Recurrent neoepitopes are also shown for the TCGA **(C)** and Ojesina et al. **(D)** cohorts. Those that were predicted for at least three patients are annotated by gene name and amino acid change.

First, using the cervical cancer samples available in TCGA CESC (13) (*n* = 194) and in the study published by Ojesina et al. (14) (*n* = 79), we proceeded to predict all potential neoantigens for this cancer type. Only genes with non-zero mRNA expression were included. Our results show that potential neoantigens are detectable in almost all samples in both studies. Only one and two patients did not have any predicted neoantigens in TCGA and Ojesina et al., respectively. The number of predicted neoantigens was from 1 to 4,049 and from 3 to 3,042 in the TCGA (Figure 1A) and Ojesina et al. (Figure 1B), respectively, and the difference in neoantigen load between the two data sets was not statistically significant. We have identified multiple recurrent mutated antigens in the TCGA (Figure 1C, MAPK1 E322K, PIK3CA E545K, PIK3CA E542K, EP300 D1399N, ERBB2 S310F, ERBB3 V104M, KRAS G12D) and also in the Ojesina et al. data set (Figure 1D, MAPK1 E322K, PIK3CA E545K, PIK3CA E542K, FBXW7 R465C). The MAPK1 E322K, PIK3CA E545K, and PIK3CA E542K mutations were found in at least three samples in both data sets. Importantly, most of the recurrent neoantigen-generating mutations are found in known oncogenic driver genes.

**Figure 2:**
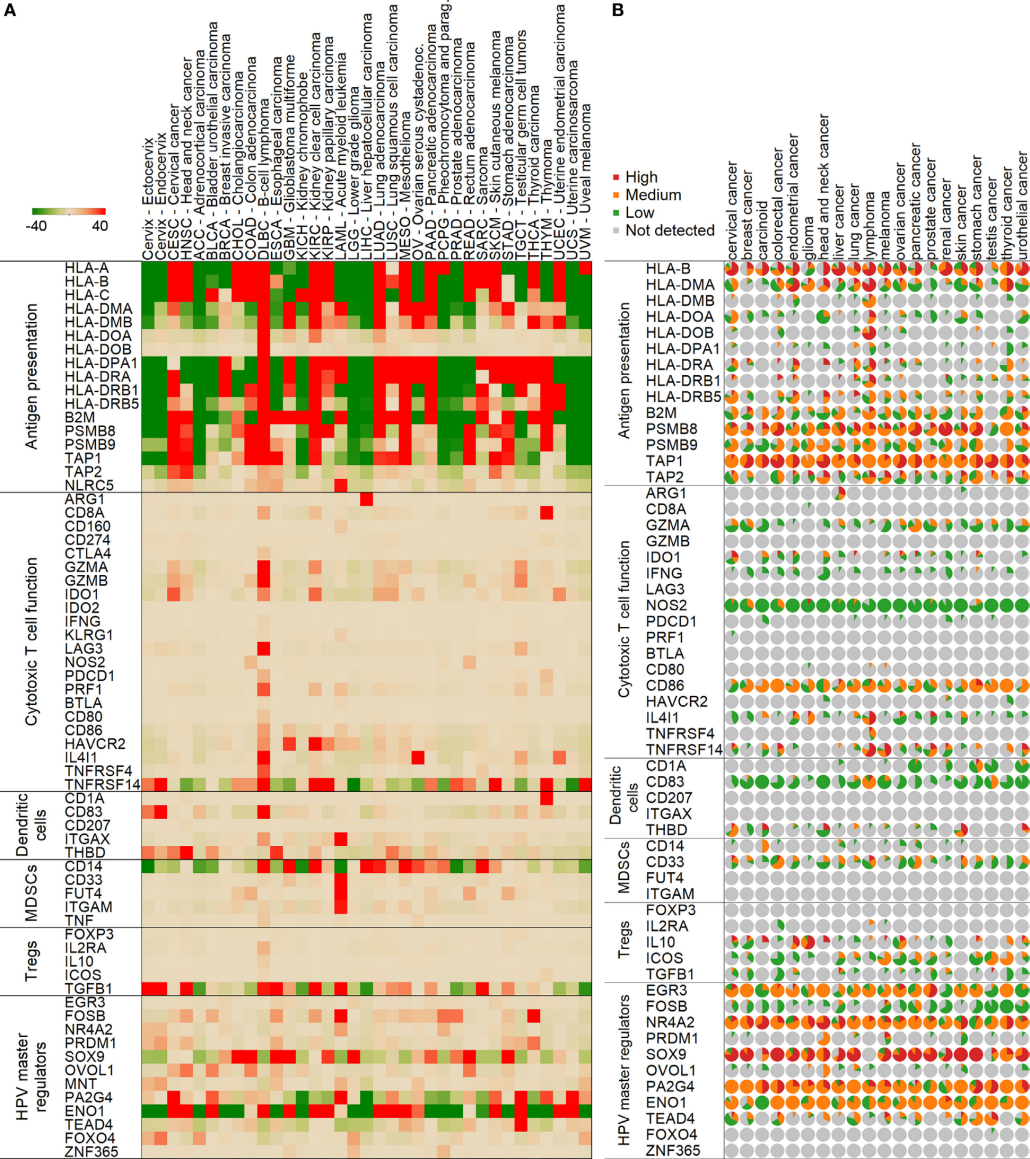
Expression of genes in normal and tumor samples mediating various immune functions. Median mRNA expressions (median centered for tissues) are represented by red (high expression) and green (low) colors **(A)**. A color scale from −40 to 40 transcripts per million was chosen to highlight differences between normal cervix and cervix cancer optimally. Normal cervix is shown in the first two columns followed by cervical cancer and other tumor types in the Cancer Genome Atlas project. Protein expressions from immunohistochemistry in the Human Protein Atlas are shown similarly **(B)**. In the pie charts, the size of the slice represents the ratio of samples with high (red), medium (orange), low (green), or no (gray) expression.

We then proceeded to look at the immunological aspect in the cervix tumor samples from both studies. Interestingly, we found that HLA class I and class II-related gene expressions that are involved in antigen presentation (HLA-A, HLA-B, HLA-C, HLA-DMA, HLA-DMB, HLA-DOA, HLA-DOB, HLA-DPA1, HLA-DRA, HLA-DRB1, HLA-DRB5, HLA-DRB6, B2M, TAP1, TAP2, PSMB8, PSMB9, and NLRC5) are generally high in cervix tumors (*n* = 306) compared to normal cervix (*n* = 11, ecto- and endocervix) and other cancers (Figure 2A). Based on the available immunohistochemistry (IHC) data on cervical tumor samples (*n* = 12) (15), most of tested tissues were positive for protein expression of HLA-B, HLA-DMA, HLA-DOA, HLA-DRA, HLA-DRB5, B2M, PSMB8, PSMB9, TAP1, and TAP2 (Figure 2B).

Importantly, the immunosuppressive enzyme IDO1 showed a significantly higher mRNA expression level in cervical cancer than in normal cervix, and also in comparison to other cancers (Figure 2A). Moreover, the protein expression of IDO1 was also found to be higher in cervical tumor samples compared to other cancers (Figure 2B). These data suggest that IDO1 may be a potential target to abrogate immune suppression.

### HPV Master Regulators Are Differentially Expressed in Cervical Cancer Compared to Normal Cervix

**Figure 3:**
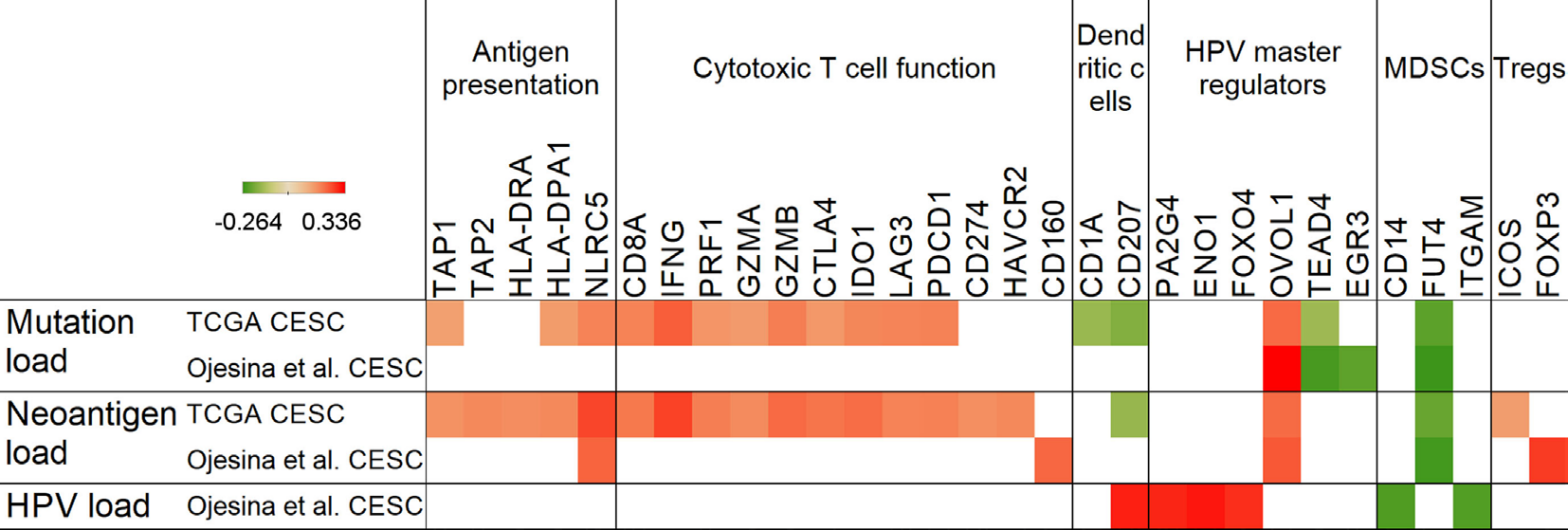
Correlation of mutation, neoantigen, and human papillomavirus (HPV) load with antigen presentation, immune markers, and master regulators. Positive Spearman’s rank correlation coefficients (red color) represent positive, while negative coefficients (green) denote negative associations with mutation, neoantigen, and HPV load in the Cancer Genome Atlas (TCGA) and Ojesina et al. data sets. Only genes with *p* < 0.05 correlations are shown.

We found that HPV E6/E7-related master regulators (ENO1, FOSB, PA2G4, SOX9, TEAD4, FOXO4, and MNT) were significantly differently expressed (*p* < 0.001) in the cervical cancer TCGA samples (*n* = 306) than in normal cervix (*n* = 11) tissue (Figure 2A). The following genes ENO1, FOSB, PA2G4, SOX9, and TEAD4 were highly expressed in cervical cancer in comparison to normal cervix (*p* < 0.001), while FOXO4 and MNT were significantly lower in cervical cancer (*p* < 0.001) (Figure 2A). According to the available data in the Human Protein Atlas (15), most of tested cervical tumor samples were positive for protein expression of EGR3, NR4A2, SOX9, PA2G4, ENO1, and TEAD4 (Figure 2B). Protein expression of FOXO4 and ZNF365 was undetectable by IHC in these samples. HPV load, which was calculated as number of HPV RNA-sequencing reads divided by library size (14), also positively correlated with expression of ENO1, PA2G4, and FOXO4 (Figure 3).

### Mutation and Neoantigen Load Show Association with Potential Immunotherapy Targets

Only a few genes (TAP1, TAP2, HLA-DRA, HLA-DPA1, and NLRC5) related to antigen presentation displayed a positive correlation with mutation or neoantigen load, and only NLRC5, a negative regulator of NF-kappaB and type I interferon signaling pathways (16), which we recently also identified as a target for immune evasion in cancer (17), showed an association with neoantigen load in both cervical cancer data sets (Figure 3). Furthermore, MDSC-associated FUT4, which encodes the CD15 protein found on neutrophils and implicated in phagocytosis, was the only immune marker, which showed a negative correlation with mutation and neoantigen load in both data sets.

The immunosuppressive molecule IDO1 (18), which was found to be overexpressed in cervical tumors (19) (Figure 2), demonstrated a positive correlation with mutation/neoantigen load in the much larger TCGA data set only (Figure 3). Other notable checkpoint-related genes that positively correlated with mutation load in the TCGA are CTLA4, PD-1, and LAG3. Interestingly, PD-L1 correlated positively with neoantigen load, but not with mutation load.

We have also identified that the OVOL1 HPV master regulator positively correlates with mutation and neoantigen load in both data sets, pointing to a potential role in cervical cancer by controlling mesenchymal–epithelial transition (20). Another master regulator, the Hippo pathway target transcription factor TEAD4, negatively correlated with mutation load in both data sets, but showed no correlation with neoantigen load. This association may be important as it has been shown that the Hippo pathway regulates cervical cancer progression (21).

### Master Regulators Show Diverse Immune Relationships and May Be Potential Targets

**Figure 4:**
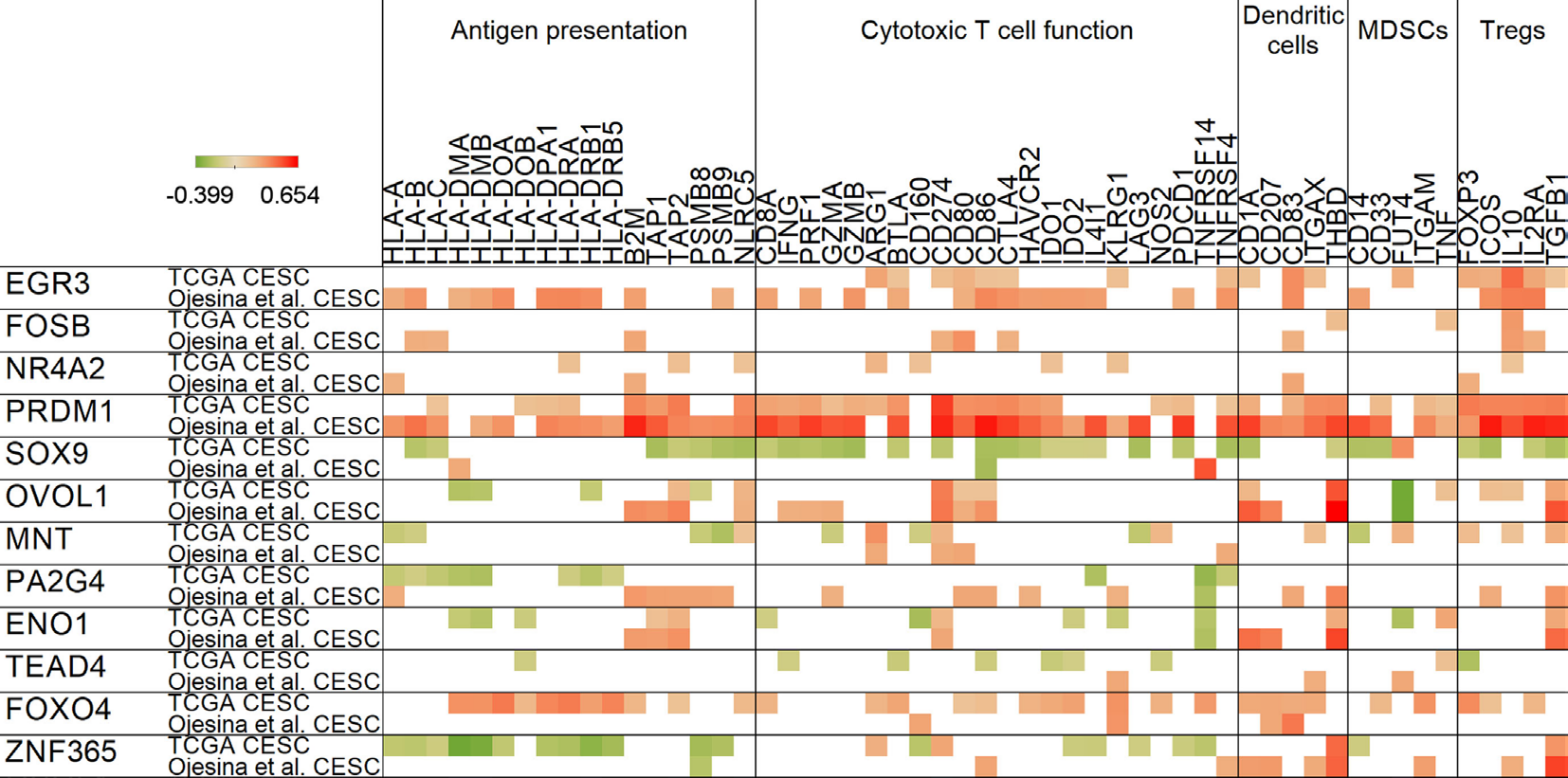
Correlation of human papillomavirus master regulator expressions with antigen presentation and immune-related genes. For all master regulators, Spearman’s rank correlation coefficients are shown in rows for both cervical cancer data sets. Red color represents positive, green denotes negative correlations. Only significant relationships (*p* < 0.05) are shown.

Our correlation analyses revealed that HPV master regulators, which are attractive targets to reverse the effects of HPV oncoproteins, show a close relationship with antigen presentation and immune markers of suppression (Figure 4). The ENO1 regulator gene, which we found to be overexpressed in cervical cancer, positively correlated with PD-L1 (CD274) and TGFB1 in both data sets, providing further indication for immunotherapy targeting in this malignancy. PD-L1 also showed a positive correlation with PRDM1, OVOL1, and MNT master regulators. Furthermore, TGFB1 expression also positively correlated with PRDM1, OVOL1, and ZNF365 expressions. ZNF365 was found to negatively correlate with expression of the PSMB8 immunoproteasome gene, highlighting another potential immunosuppressive mechanism by HPV master regulators.

NF-κB inhibitor FOXO4 (22) was the only master regulator that showed an association with the KLRG1 T cell activation maker often associated with senescence, exposing again a potential immunosuppressive mechanism. Expression of FOXO4 also positively correlated with HPV load (in Figure 3). Another notable positive correlation we identified was between the immunosuppressive cytokine IL10 and master regulators EGR3 and FOSB. Since it was reported that IL10 plays a role in maintenance of Tregs and immunosuppression in cervical cancer, this correlation might be important in understanding the mechanism (23).

## Discussion

Antigen presentation plays a crucial role in human host defense, including immune response to cancer. Immunotherapies are often based on targeting antigens presented *via* major histocompatibility complex/HLA molecules (24). Tumors display tumor-associated antigens on class I and class II HLA molecules at the cell surface, and these antigens can be recognized by CD8 and CD4 T cells. Adoptive T cell therapy uses *ex vivo* expanded tumor-specific T cells that are infused to the patient to elicit tumor regression. This strategy has shown very promising results in the clinic using HPV-specific tumor-infiltrating lymphocytes (6). However, it is clear that the currently available approach alone will not be sufficient to cure cervical cancer. Exome sequencing of a large set of cervical carcinomas revealed an average of 99 missense mutations per sample (14). To determine if these mutations could be targets for individualized T cell immunotherapy, we have evaluated the availability and potential immunogenicity of mutated antigens (i.e., neoantigens) that are predicted to bind HLA in two large cohorts of cervical cancer patients.

We found that most tumors have predicted neoantigens that are capable of binding the HLA molecules of the patient. However, immunogenicity of mutated antigens will need to be validated extensively before clinical application. It was shown by multiple studies that only a minority of predicted neoantigens are immunogenic (25–27). In the case of a set of predicted Cytomegalo-, Epstein–Barr-, and Influenza virus peptides, about half of all peptides generated a T cell response detectable by IFN-γ production (28). However, it was recently shown that healthy donors may provide a source of neoantigen-specific T cells even when the autologous tumor-infiltrating lymphocytes do not react to the predicted neoantigens (29). Using this strategy greatly increases the chance for developing successful immunotherapies using neoantigens that we identified. Therefore, our data suggest that among the thousands of predicted neoantigens per patient, there may be multiple immunogenic neopeptides that could be effective targets, including those that are derived from recurrent mutations. A few of these recurrent neoantigens are from known oncogenic drivers, for example, PIK3CA, MAPK1, ERBB2, ERBB3, and also from the KRAS G12D mutation, which we recently identified as a promising recurrent neoantigen-based immunotherapy target in pancreatic ductal adenocarcinoma (30). As it was shown in metastatic colorectal cancer, the KRAS G12D mutation can be successfully targeted by specific tumor-infiltrating lymphocytes (31).

Our study also determined a relationship between HPV master regulator genes and antigen presentation and cytotoxic and suppressive immune activity (12). We highlight the duality of the presence of an immune response and the establishment of multiple suppression mechanisms occurring in direct connection to the immune response. The OVOL1 master regulator positively correlated with mutation and neoantigen load, and it was also associated with higher TGFB1 expression. TGF-beta is a well-studied cytokine and immunosuppressive molecule, which has been shown to affect MHC expression (32, 33) and inhibits the ability of dendritic cells to present antigen to stimulate T lymphocytes (34). In addition, OVOL1 also represses c-Myc transcription (35), and as c-Myc level is a known poor prognostic factor in cervical cancer (36). Therefore, OVOL1 may play a complex role in regulating the growth and progression of cervical cancer. Other master regulators, namely PRDM1, ENO1, and ZNF365 showed positive correlations with TGFB1. In the Human Protein Atlas, OVOL1 and PRDM1 showed low, ENO1 medium, while ZNF365 showed no protein expression in cervical cancer (15).

The FOSB gene, which has been shown to be involved in CD95L-initiated apoptosis of T cells (37), was associated with expression of the IL10 immunosuppressive cytokine. FOSB is part of the activator protein-1 (AP-1) complex, and it was previously reported that repression of AP-1 activity and HPV transcription may be effective in controlling cervical tumors (38). FOSB expression is also significantly higher in cervical cancer compared to normal cervix; therefore, it may be a potential target to help overcome immune suppression. EGR3 also showed a positive correlation with IL10 in both data sets. This may be important because EGR3 is a key negative regulator of T cell activation (39). Protein expression data from the Human Protein Atlas indicates low to medium FOSB, and medium to high EGR3 expression.

Another master regulator that potentially plays a role in immune evasion is ENO1. This gene is overexpressed in cervical cancer, and its expression positively correlates with HPV load, and also PD-L1 and TGFB1 expression. Overexpression of the encoded protein ENOA has been detected in several cancers, and ENOA is also able to induce immune response and shows clinical correlations in cancer patients; however, no HLA class I-restricted ENOA peptide has been identified (40). The Human Protein Atlas also shows medium ENO1 expression in cervical cancer. Furthermore, ENO1 is a prognostic marker in the HPV-associated head and neck cancer (41), and it was recently shown that ENO1 silencing impairs cancer cell line growth (42). Based on our results and these previous studies on the role of ENO1 in tumor progression and immune response, we propose that targeting ENO1/ENOA in cervical cancer will provide an additional therapeutic benefit and increase the patient survival.

In conclusion, this study highlights the relationships between the expression of HPV oncoprotein-associated master regulators, neoantigen landscape, the mutation load, and the immune activity in cervical cancer. Analysis of this complicated network and probing of potential interactions will provide attractive targets to increase effectiveness of immunotherapies. In addition to further validation of these targets at the protein level, further *in vitro* and *in vivo* studies will be needed to confirm the regulatory effect of these HPV mater regulators on the expression of major immunosuppression markers in cervical tumors. Moreover, mass spectrometry-based peptide/antigen identification will also be necessary for their use as immunotherapy targets.

## Materials and Methods

### Exome and RNA-Sequencing Data

Mutation, gene expression, and clinical data from the cervical cancer TCGA were obtained from public TCGA repositories and from the publication of Ojesina et al. (14). Raw exome-sequencing data (.bam files) that we used to perform HLA typing were obtained though dbGaP (https://www.ncbi.nlm.nih.gov/gap). HPV load (number of HPV RNA-sequencing reads divided by library size) was available in a supplementary file of the Ojesina et al.’s (14) publication.

### Neoantigen Prediction

All 8–12-mer wild type and mutated neopeptides and their HLA-binding affinities were predicted for HLA-A, -B, -C alleles as it was described earlier (43). We used all missense mutations downloaded from the cervical cancer (CESC) TCGA (13) and Ojesina et al. (14) projects. To determine peptide-binding affinities to HLA-A, -B, and -C alleles, we used the NetMHCpan (version 2.8) (44) program, which applies artificial neural networks to predict peptide-MHC class I binding. HLA types of patients were predicted from the raw exome-sequencing data of normal and tumor samples using the Athlates (version 2014_04_26) (45) HLA type prediction software. A peptide was considered a strong binder if the predicted HLA binding affinity was <50 nM, and it was regarded as a weak binder if the HLA-binding affinity was between 50 and 500 nM.

### Correlation and Other Statistical Analyses

We calculated all Spearman’s rank correlation coefficients using the R software. For comparisons of two groups, we used two-tailed Student’s *t*-tests. Differences were considered significant when *p* < 0.05.

## Author Contributions

Study concept and design: JR. Acquisition, analysis, or interpretation of data: YQ, SE, M-AF, LS, PH, EG, AJ, and JR. Preparation, review, or approval of the manuscript: YQ, SE, M-AF, LS, PH, EG, AJ, and JR.

## Conflict of Interest Statement

The authors declare that the research was conducted in the absence of any commercial or financial relationships that could be construed as a potential conflict of interest.
